# Metabolites Involved in Purine Degradation, Insulin Resistance, and Fatty Acid Oxidation are Associated with Prediction of Gestational Diabetes in Plasma

**DOI:** 10.1007/s11306-021-01857-5

**Published:** 2021-11-27

**Authors:** Lauren E. McMichael, Hannah Heath, Catherine M. Johnson, Rob Fanter, Noemi Alarcon, Adilene Quintana-Diaz, Kari Pilolla, Andrew Schaffner, Elissa Jelalian, Rena R. Wing, Alex Brito, Suzanne Phelan S, Michael R. La Frano

**Affiliations:** 1 Department of Food Science and Nutrition, California Polytechnic State University, San Luis Obispo, CA, USA; 2 College of Agriculture, Food and Environmental Sciences, California Polytechnic State University, San Luis Obispo, CA, USA; 3 Cal Poly Metabolomics Service Center, California Polytechnic State University, San Luis Obispo, CA, USA; 4 Department of Kinesiology and Public Health, California Polytechnic State University, 1 Grand Ave, San Luis Obispo, CA, 93407, USA; 5 Center for Health Research, California Polytechnic State University, San Luis Obispo, CA, USA; 6 Department of Statistics, California Polytechnic State University, San Luis Obispo, CA, USA; 7 Department of Psychiatry and Human Behavior, Warren Alpert Medical School at Brown University, Providence, RI, USA; 8 Laboratory of Pharmacokinetics and Metabolomic Analysis. Institute of Translational Medicine and Biotechnology. I.M. Sechenov First Moscow Medical University, Moscow, Russia; 9 World-Class Research Center “Digital Biodesign and Personalized Healthcare”, I.M. Sechenov First Moscow State Medical University, Moscow, Russia

**Keywords:** Gestational Diabetes Mellitus, Metabolomics, Lipidomics, Metabolites, Pregnancy

## Abstract

**Introduction:**

Gestational diabetes mellitus (GDM) significantly increases maternal and fetal health risks, but factors predictive of GDM are poorly understood.

**Objectives:**

Plasma metabolomics analyses were conducted in early pregnancy to identify potential metabolites associated with prediction of Gestational Diabetes Mellitus (GDM).

**Methods:**

Sixty-eight pregnant women with overweight/obesity from a clinical trial of a lifestyle intervention were included. Participants who developed GDM (n=34; GDM group) were matched on treatment group, age, body mass index, and ethnicity with those who did not develop GDM (n=34; Non-GDM group). Blood draws were completed early in pregnancy (10-16 weeks). Plasma samples were analyzed by UPLC-MS using three metabolomics assays.

**Results:**

One hundred thirty moieties were identified. Thirteen metabolites including pyrimidine/purine derivatives involved in uric acid metabolism, carboxylic acids, fatty acylcarnitines, and sphingomyelins (SM) were different when comparing the GDM vs. the Non-GDM groups (p<0.05). The most significant differences were elevations in the metabolites’ hypoxanthine, xanthine and alpha-hydroxybutyrate (p<0.002, adjusted p<0.02) in GDM patients. A panel consisting of four metabolites: SM 14:0, hypoxanthine, alpha-hydroxybutyrate, and xanthine presented the highest diagnostic accuracy with an AUC= 0.833 (95% CI: 0.572686-0.893946), classifying as a “very good panel”.

**Conclusion:**

Plasma metabolites mainly involved in purine degradation, insulin resistance, and fatty acid oxidation, were altered in early pregnancy in connection with subsequent GDM development.

## Introduction

Gestational Diabetes Mellitus (GDM) manifests as hyperglycemia in a mother who has not previously had diabetes (CDC, 2019) and affects up to 10% of pregnancies in the US each year (CDC, 2019). According to estimations of the American Diabetes Association (ADA), GDM costs $1.29 billion annually in the United States (Dall et al., 2014). Age, ethnicity, geographical location, and modifiable lifestyle factors are among the risk factors that elevate the likelihood of developing the disease during gestation (McIntyre et al., 2019). Obesity, previous family history of GDM, and glycosuria are among the factors most highly associated with an increased risk of GDM (“Gestational Diabetes Mellitus,” 2004). Although some level of insulin resistance is expected as a natural occurrence in all pregnancies, GDM is a pathological state characterized by inadequate insulin production leading to hyperglycemia (Buchanan et al., 2007; Dahlgren, 2006). The mechanism for developing the disease is still unknown, but it is apparent that the elevation in diabetogenic hormones during pregnancy can negatively impact the secretion of insulin by the beta-cells in the pancreas (Buchanan et al., 2007; Dahlgren, 2006; McIntyre et al., 2019). GDM increases the risk of a plethora of complications for the mother and the offspring pre- and post-partum (Catalano et al., 2003), including macrosomia, premature birth, respiratory distress syndrome, neonatal hypoglycemia, and a higher risk of developing type 2 diabetes mellitus and cardiometabolic diseases later in life (Mitanchez et al., 2015). In addition to these complications, patients with GDM must manage their blood glucose levels during pregnancy primarily by dietary modifications and exercise interventions, and the possible addition of pharmacotherapy including insulin if normoglycemia is not achieved (CDC, 2019).

The use of intervention strategies to decrease GDM’s severity often come later in pregnancy after the mother has been diagnosed. Intervention strategies include a low-glycemic-index diet, increased physical activity, and, if necessary, insulin administration or oral hypoglycemic drugs (McIntyre et al., 2019). Excess weight gain during pregnancy has been shown to be related to GDM development, but prenatal interventions to reduce excess gestational weight gain have had minimal effects on GDM prevention. Healthy Beginnings, a randomized control trial, found lifestyle intervention to be effective in reducing excess gestational weight gain, but had no effect on GDM development (Phelan et al., 2018). Similarly, previous randomized control trials have found prenatal intervention strategies, ranging from dietary intervention to physical activity, to be largely ineffective in reducing GDM occurrence (Han et al., 2012; Tieu et al., 2017).

Further research regarding factors predictive of GDM is necessary to improve the timing and method of GDM prevention interventions. Metabolomics, the extensive measure of small molecules in a biological sample, can be a useful tool in identifying metabolites for early prediction of GDM among high-risk pregnant women (Dettmer et al., 2007). This would provide insight into mechanisms of GDM development that could inform the development of novel prevention or intervention methods (Landon et al., 2009). The objective of the study is to use metabolomics analyses to identify first-trimester plasma metabolites that may be associated with early prediction of GDM.

## Participants and Methods

### Study Design

This was a secondary analysis using samples from the Healthy Beginnings/Comienzos Saludables Study, a randomized clinical trial (RCT) that is part of the Lifestyle Interventions for Expectant Moms (LIFE-Moms) Consortium (Phelan et al., 2018). This RCT focused on the outcomes of behavior lifestyle change on weight gain during gestation (Phelan et al., 2018). Samples were acquired from two study sites: California Polytechnic State University, San Luis Obispo, California and Miriam Hospital with Women and Infants Hospital in Providence, Rhode Island. This trial was registered as NCT01545934. Eligibility consisted of being 9-16 weeks gestational age, BMI (in kg/m2) ≥25 upon study entry height and weight, English or Spanish speaking, age ≥18 years old, and singleton pregnancy. Participants were excluded if glycated hemoglobin ≥6.5, reported major health diseases, substance abuse, undergoing treatment for serious psychological disorders, had contradictions to aerobic exercise, or who had repeated no-shows or loss of contact during screening. The fasting blood draw used for metabolomics analysis was collected prior to randomization. These blood draws were processed centrally following identical procedures established prior to study initiation at each site and conducted by certified staff. Participants were randomly assigned to two different intervention methods. Treatment group one received enhanced usual care, which represented the control group. This group received all aspects of usual care offered by their prenatal care providers, as well as attendance of an ~20-min welcome visit with a study interventionist that provided general information about healthy eating, physical activity, and the IOM recommendations for total gestational weight gain. Treatment group two had a multi-component lifestyle intervention, which included diet, exercise, and behavioral change. This group received enhanced usual care, in addition to a behavioral lifestyle intervention designed to prevent excessive weight gain during pregnancy. This included a meal replacement plan, were directed to aim for 30 min of activity on most days of the week, and had face-to-face counseling sessions with a study interventionist every 2 wk until 20 wk of gestation and then monthly visits until delivery but went more often if gestational weight gain exceeded ~0.5 pounds/wk. More information concerning treatments is detailed in Phelan et al. 2018 (Phelan et al., 2018). Data was collected throughout pregnancy, including blood samples, diet assessment, and clinic measured GDM diagnosis. Blood samples were taken between gestational weeks 10-16. Since the multi-component lifestyle intervention showed no statistically significant effect on GDM occurrence (p=0.7) (Phelan et al., 2018), the samples used for this secondary analysis are from both the control and treatment groups. There were a total of 34 GDM cases that were collected from the California (n=13) and Rhode Island (n=21) study sites. Samples for 34 GDM cases were matched to 34 healthy controls prior to metabolomics analysis based on age, study entry body mass index (BMI), ethnicity, study site, and treatment. The two groups did not differ in weight gain from entry to 26 weeks, nor did they differ from entry to 35 weeks.

### Dietary intake assessment

Participants’ dietary intake was collected through a 24 hour recall at two random days of the week at the beginning of the study (Phelan et al., 2018). The National Cancer Institute Automated Self-Administered 24 hour recall (ASA-24) was used to assess diets (Kirkpatrick et al., 2014). Total daily energy, macronutrient, and micronutrient intake were included in the assessment (Subar et al., 2012).

### Metabolomics analyses

Samples were randomized, de-identified, and assigned new IDs for processing and analysis. Targeted metabolomics assays for primary metabolomics, aminomics, and lipidomics were performed on plasma samples using protein precipitation extraction with ultra-performance liquid chromatography tandem quadrupole mass spectrometry (UPLC-MS) using modified previously published methods (La Frano et al., 2020). Briefly, 25 μL of plasma were added to 1.5 mL tubes before the addition of 10 μL of 1 μM internal standard solution, followed by 750 μL chilled methanol. Samples were then vortexed 30 seconds prior to being centrifuged at 15,000 x G for 10 min. The supernatant was transferred to 1.5 mL high performance liquid chromatography (HPLC) amber glass vials, dried by centrifugal vacuum evaporation, and reconstituted in 100 μL 3:1 acetonitrile:methanol solution with 1-cyclohexylureido, 3-dodecanoic acid (CUDA; Sigma-Aldrich, St. Louis, MO, USA) solution. The reconstituted solution was vortexed 30 seconds and placed on ice for 10 minutes. The solution was then centrifuged at 10,000 x G for 3 minutes after being transferred to microfilter tubes. The supernatant was then transferred to a HPLC vial to be analyzed using the UPLC-MS. All UPLC-MS analyses, including primary metabolomics, aminomics, and lipidomics, were conducted on a Waters Acquity I-Class UPLC (Waters, Milford, MA, USA) coupled with an API 4000 QTrap (Sciex, Framingham, MA) and quantified with AB Sciex MultiQuant version 3.0 in order to generate peak area values. Primary metabolomics and aminomics used multiple reaction monitoring (MRM) while the lipidomics assay used full scan MS over m/z 400-1000 using Q1 scans at unit mass resolution and specific lipid species were identified using a range to capture the full width of the monoisotopic ion, as previously described (Townsend et al., 2013). For the primary metabolomics assay, metabolites were separated using a 150 X 2.0 mm Luna NH2 column (Phenomenex, Torrance, CA) and detected by negative ion mode electrospray ionization. For the aminomics assay, metabolites were separated using a 150 × 2.1 mm Atlantis HILIC column (Waters) and detected by positive ion mode electrospray ionization. For the lipidomics assay, metabolites were separated using a 150 × 3.0 mm Prosphere HP C4 column (Grace, Columbia, MD, USA) and detected by positive ion mode electrospray ionization. The primary metabolomics assay screened for 80 metabolites, including 23 carboxylic acids, 23 purines/pyrimidines/nucleotides/nucleosides, 14 carbohydrates, 12 amino acid derivatives, five sterols, two vitamins, and several fatty acid derivatives, glycerophospholipids, peptides, and phenols. The aminomics assay screened a total of 83 metabolites, including 23 acylcarnitines, 20 amino acids, 12 amino acid derivatives, eight purines/pyrimidines/nucleotides/nucleosides, eight carboxylic acids, three quaternary ammonium compounds, two carbohydrates, and several imadazoles, heterocyclic compounds, glycerophospholipids. Lastly, the lipidomics assay screened a total of 51 metabolites, including 18 phosphatidylcholines, eight lysophosphatidylcholines,, four phosphatidylethanolamines, six lysophosphatidylethanolamines and 15 sphingomyelins (SM).

Primary metabolomics and aminomics assay metabolite identities were confirmed using pure standards in order to establish retention time and MRM, as well as optimize instrument parameters. Standards included those within the Mass Spectrometry Metabolite Library of Standards (MSMLS; Sigma-Aldrich), as well as individually purchased standards from Sigma-Aldrich, Cambridge Isotope Laboratories, Inc (Tewksbury, MA, USA). and Cerilliant Corporation (Round Rock, TX). Some acylcarnitine species were identified based on MRM only, as noted in Online Resource Table 1. For the lipidomics assay, the SPLASH® LIPIDOMIX® Mass Spec Standards purchased from Avanti Polar Lipids Inc. (Alabaster, AL, USA) were utilized to identity select lipid species retention times in order to establish retention time indexes (Zheng et al., 2018) that adjust for retention time differences in specific lipid species between our method and those provided by Townsend et al. (Townsend et al., 2013). Purified egg yolk extracts (99-97% purity by TLC; Sigma-Aldrich) of each targeted lipid class, consisting of a variety of species, were analyzed in order to confirm approximate retention time ranges for phosphatidylcholines, phosphatidylethanolamines, lysophosphatidylcholines, lysophosphatidylethanolamines, and sphingomyelins. Surrogate standards used in the primary metabolomics assay included succinate-^13^C_4_, sorbitol-1,1,2,3,4,5,6,6-d8, octanoate-^13^C_8_, adenine-2-d1, and histamine-α,α,β,β-d4 while the aminomics assay used L-tryptophan-^13^C_11_, adenine-2-d1, 2-(3,4-Dihydroxyphenyl)ethyl-1,1,2,2-d4-amine, and histamine-α,α,β,β-d4. These were utilized to monitor extraction efficiency and recovery percentage for each sample analyzed. Surrogates were purchased from Santa Cruz Biotechnology, Inc, (Dallas, TX, USA), CDN Isotopes Inc. (Pointe-Claire, Quebec, Canada), and Cambridge Isotope Laboratories, Inc. The internal standard CUDA (Sigma Aldrich), included in the reconstitution solvent that was added post-extraction, was used for controlling instrument and injection parameters. All primary metabolomics and aminomics raw data were normalized to the internal standard CUDA (Sigma-Aldrich). The lipidomics assay raw data were normalized to the mTIC.

For quality control purposes, compounds whose background (as determined by method blank response) was greater than 50% of the average sample response or that had more than 1/3 of samples with signal to noise less than 3:1, were excluded from the dataset. To assess reproducibility, five replicates of the current study samples were separately extracted and analyzed. A pooled plasma sample collected from a different study was used as a long-term reference QC sample for an inter-study assessment of data. All samples were run in a single batch.

### Statistical analyses

Comparisons of the metabolites between the GDM and no-GDM was performed by fitting a mixed model with fixed effects GDM, covariates adjusting for age, study entry BMI, treatment (control or intervention group), and ethnicity, and random effects study site, study ID, and matched case-control pair ID in JMP Pro 14.0.0 (SAS Institute Inc., Cary, NC) (Pearce, 2016). Prior to analysis, data were assessed using the Shapiro-Wilk test, with normally distributed data analyzed raw while non-normally distributed data were log-transformed. Multiple comparisons were adjusted for using the Benjamini-Hochberg procedure (Benjamini & Hochberg, 1995) with a cutoff of 0.05 to control the false discovery rate (FDR). Multivariate analysis were performed on covariate-adjusted data (age, study entry BMI, treatment, and ethnicity). Multivariate analysis was conducted using principal component analysis (PCA) and partial least squares discriminant analysis (PLS-DA) in MetaboAnalyst 4.0 (http://www.metaboanalyst.ca/, Canada) (Xia & Wishart, 2016). MetaboAnalyst was also used for predictive models created after evaluating different combinations of metabolites using linear SVM ROC curve analysis with Monte-Carlo cross validation with 95% confidence intervals (CI) (Xia et al., 2013). K-means (KM) clustering was performed to detect features with similar behavior. The diagnostic accuracy was determined for metabolites by calculating the areas under the curve (AUCs) by contrasting the non-GDM versus the GDM groups. The areas under the curve (AUC) were categorized as “test not useful”, “bad”, “sufficient”, “good”, “very good” or “excellent” if AUCs were < 0.5, 0.5–0.6, 0.6–0.7, 0.7–0.8, 0.8–0.9 or 0.9–1.0, respectively (šimundić, 2009). Performance was measured by assessing predicative accuracy using 100 permutations.

All raw data and metadata are available through the NIH Metabolomics Workbench under Study ID: ST001948 (Sud et al., 2016).

## Results

### Participant characteristics, cardiometabolic risk factors, and diet analysis

The GDM and healthy control groups were not different in age, BMI or ethnicity (Table 1). Prior to metabolomics analysis, numerous circulating cardiometabolic risk factors collected during first trimester were tested in the study participants (Table 1). Total fasting triglycerides was significantly higher while HDL cholesterol was lower in the GDM group (p<0.05). Fasting glucose, insulin, HOMA-IR, total cholesterol, LDL cholesterol, leptin, and C-Peptide were not different between groups. Based on 24-hour dietary recall data at study entry, sodium intake was higher in the GDM group than in non-GDM group (p<0.05; Online Resource Table 2). The estimated intake of energy, percentages of macronutrients consumed, total macronutrients, and micronutrients were not different between groups.

### Univariate Analysis

A total of 130 metabolites were detected in the samples, including 41 primary metabolomics, 72 aminomics, and 16 lipidomics assay metabolites. The GDM effect in the mixed models identified 13 metabolites that were different (p<0.05) between the GDM and non-GDM cases in the first trimester samples (Table 2). These included metabolites belonging to the classes of purines and pyrimidines and their derivatives, carboxylic acids, amine derivatives, acylcarnitines, and SMs. These moieties have been described to be mainly involved in purine degradation, fatty acid oxidation, mitochondrial function, the kynurenine pathway, and sphingolipid metabolism (Koves et al., 2008; Oxenkrug, 2013; Roszczyc-Owsiejczuk & Zabielski, 2021; Zhao et al., 2019) . The most significant differences between the groups were the metabolites’ xanthine, hypoxanthine, and alpha-hydroxybutyrate (p<0.002), with all having significant FDR-adjusted p-values (adjusted p<0. 02). These compounds were elevated in GDM cases as well as the beta-hydroxybutyrate, palmitoylcarnitine, malonylcarnitine, kynurenine, nicotinamide, SM C15:0, SM C16:1, SM C22:0, and uridine (p<0.05). A summary of the metabolomics results can be viewed in Online Resource Table 1.

### Multivariate analysis

Multivariate analysis using PCA did not distinguish groups (Online Resource Figure 1). However, when PLS-DA was used to distinguish the GDM group from the healthy controls, the cross validation indicated the fraction of the variance that the model explains in the independent (X) and dependent variables (Y) were R^2^=0.52 and Q^2^=0.09 with a 0.66 accuracy in a two component model, respectively. The predictive accuracy of the model as measured by 100 permutations was significant (p=0.04). Although separation between groups in PLS-DA was better than PCA, it did not distinguish all samples (Online Resource Figure 2). The top five single metabolites identified using ROC curve analysis were hypoxanthine, alpha-hydroxybutyrate, xanthine, SM 14:0, and SM 15:0 presenting “good” diagnostic accuracy (Table 2). A panel consisting of four metabolites: SM 14:0, hypoxanthine, alpha-hydroxybutyrate, and xanthine presented the highest diagnostic accuracy with an AUC= 0.833 (95% CI: 0.686-0.946), classifying as “very good panel” (Online Resource Figure 3). Predictive accuracy for this combined panel was significant (p = 0.004).

## Discussion

The present study utilized a combined metabolomics approach of three assays to measure known metabolites in plasma collected during the first trimester for women who later developed GDM and compared it with women who did not develop GDM. This study represents one of few studies that have collected first trimester samples in order to identify potential metabolites associated with GDM risk. Metabolites that were significantly different between GDM and non-GDM groups at first trimester are mainly related to purine degradation, insulin resistance, and fatty acid oxidation.

**Fig. 1:**
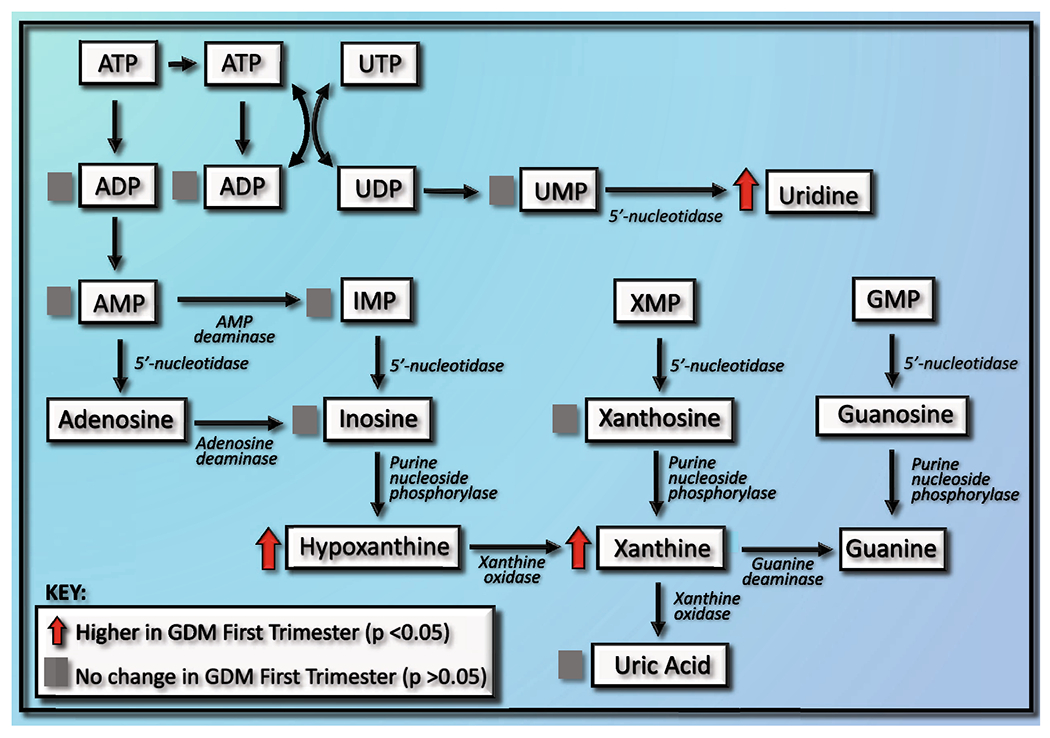
Proposed intersecting pathways related to purine and pyrimidine degradation altered at first trimester in women that develop gestational diabetes mellitus

Hypoxanthine is a breakdown product of ATP and has been shown to be associated with elevated ATP loss and energy imbalance (Gerber et al., 2014). Another study found hypoxanthine to be significantly different between GDM and Non-GDM groups (Zhao et al., 2019). They found that hypoxanthine was elevated in GDM groups only in the first trimester (Zhao et al., 2019). They concluded that the increased level during first trimester could be due to an assumed increased energy intake of women with GDM (Zhao et al., 2019). However, based on the 24-hour dietary recalls performed in the study described herein, we did not observe differences in energy intake between groups. This may suggest that hypoxanthine’s change in concentration may be due to its role in uric acid metabolism (Figure 1) (Zhao et al., 2019). As part of purine degradation, xanthine oxidase (XO) catalyzes the breakdown of hypoxanthine to xanthine and xanthine to uric acid (Law et al., 2017)(Figure 1). Since uric acid has been related to insulin resistance, high levels of hypoxanthine may increase levels of uric acid and therefore possibly lead to GDM (Yamamoto et al., 1997). In fact, uric acid levels have been associated with GDM development in multiple previous studies (C et al., 2014; Pleskacova et al., 2018). However, uric acid levels were not different between the Non-GDM and GDM groups in this study. Therefore, it is possible there is progressive dysregulation of this pathway over the course of GDM development that would eventually change uric acid levels. The elevated levels of the pyrimidine uridine in GDM patients may be due to pyrimidine degradation. Altered uridine levels have been associated with ATP depletion and mitochondrial dysfunction (Zhang et al., 2020). Future research is needed to address the possible interaction between ATP depletion, purine and pyrimidine degradation, and GDM development.

**Fig. 2:**
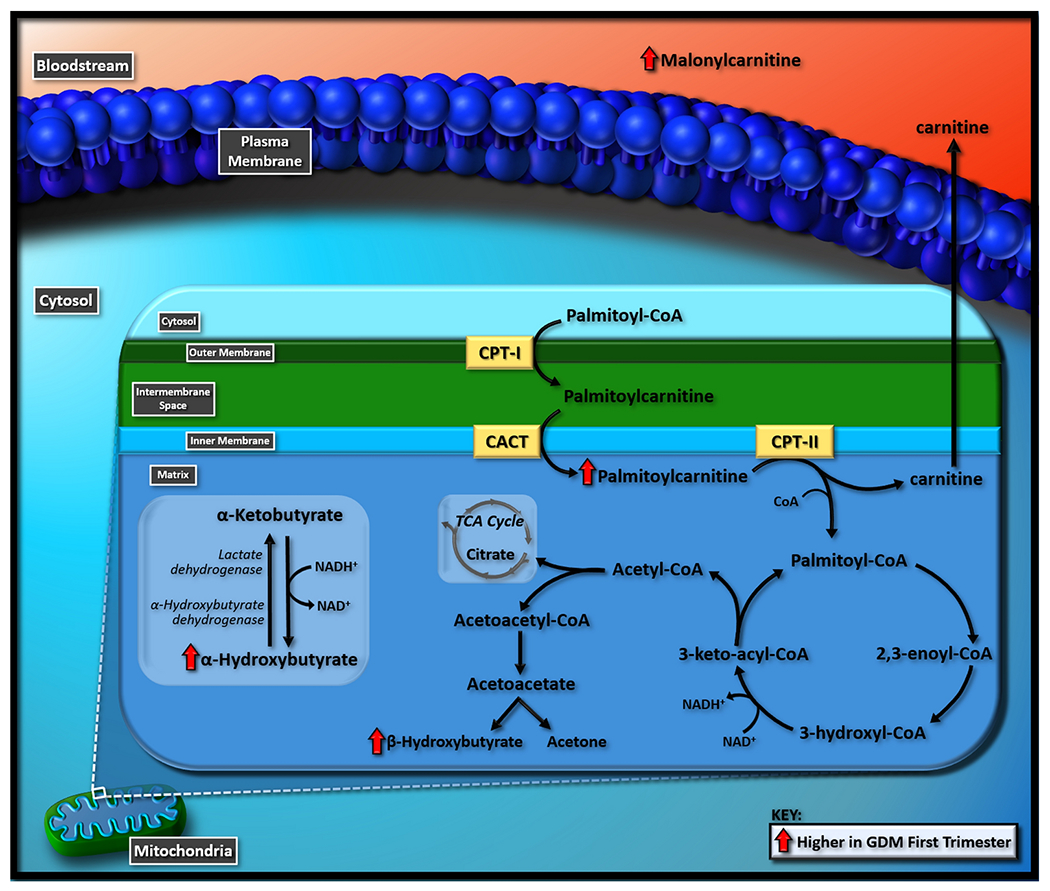
Proposed intersecting pathways related to fatty acid oxidation altered at first trimester in women that develop gestational diabetes mellitus

Insulin resistance alters the body’s metabolism and further, the type and amount of compounds present in the blood. Due to the decreased ability of glucose to be utilized for energy, the body compensates by using energy from fat. This increases the amount of fatty acid oxidation, which alters the body’s metabolic profile. As a byproduct of excess NADH + H^+^ produced from increased fatty acid oxidation, alpha-hydroxybutyrate may be produced from alpha-ketobutyrate (Gall et al., 2010; Sharma et al., 2021)(Figure 2). Previous research identified alpha-hydroxybutyrate as a metabolic marker for insulin resistance (Cobb et al.. 2013). In fact, one study found alpha-hydroxybutyrate to be directly correlated with insulin resistance and dysfunction in glucose regulation in individuals without underlying health conditions (Gall et al., 2010). Additionally, alpha-hydroxybutyrate was found to be elevated in GDM cases in the second and third trimesters (Dudzik et al., 2017).

In support of alpha-hydroxybutyrate being a consequence of increased fatty acid oxidation were the observed increases in GDM patients of beta-hydroxybutyrate, palmitoyl-carnitine, and malonyl-carnitine (Figure 2), as well as differential changes in multiple other acylcarnitines. Beta-hydroxybutyrate is a ketone body, a product of fatty acid oxidation, and is often utilized to diagnose diabetic ketoacidosis (Sheikh-Ali et al., 2008). Elevated levels of beta-hydroxybutyrate in our study could be evidence of an increase in fatty acid oxidation and show the progression of GDM as early as the first trimester. It has been found to be 2.5 times higher in mothers with diabetes at week 6 of gestation (Jovanovic et al., 1998) and has also been elevated in GDM cases during the second and third trimesters (Dudzik et al., 2017). The role of palmitoyl-carnitine is to facilitate the transportation of the long-chain fatty acid palmitate across the inner mitochondrial membrane and into the mitochondrial matrix of the cell, where oxidation occurs. Research suggests that palmitoyl-carnitine can be used as a marker for fatty acid oxidation (Dudzik et al., 2017) and has also been elevated in GDM cases in first trimester in a similar study (Zhao et al., 2019). The increase in malonyl-carnitine may be reflective of an increase in malonyl-CoA, which increases fatty acid synthesis while inhibiting fatty acid oxidation via the inhibition of CPT1, an enzyme involved in the transport of fatty acyl-CoAs into the mitochondria for fatty acid oxidation (Liempd et al., 2021). This increase in malonyl-CoA may cause acylcarnitine concentrations to decrease, thus resulting in an increase in plasma long-chain fatty acids and acyl-CoAs later in pregnancy. Elevated long-chain fatty acids have been observed in multiple third trimester GDM studies (X. Chen et al., 2010; Dudzik et al., 2017; Pinto et al., 2015). While alterations in acylarnitines are mixed among third trimester GDM studies, Dudzik et al.’s post-diagnosis study found acylcarnitines, particularly long-chain acylcarnitines, to be lower in the GDM compared to the non-GDM group (Dudzik et al., 2014). However, fatty acids, malonyl-CoA, and other acyl-CoAs were not measured in this study, so further research is necessary to confirm this occurrence.

The higher levels of kynurenine and nicotinamide observed in GDM patients may suggest an upregulation in the kynurenine pathway. Alterations in the kynurenine pathway have been observed in multiple type 2 diabetes mellitus studies, possibly due to inflammation-induced upregulation of enzymes involved in converting tryptophan to kynurenine (Oxenkrug, 2013). An increase in kynurenine is associated with an increase in HOMA-IR in type 2 diabetes mellitus, and is also correlated with quinolinic acid, a downstream metabolite of the kynurenine pathway that is positively associated with type 2 diabetes mellitus incidence (Yu et al.. 2018).

The decrease in SMs’ 14:0, 15:0, 16:1, and 22:0 indicates dysregulated sphingolipid metabolism. Past early-pregnancy GDM studies have noted SMs to be lower in GDM cases compared to non-GDM cases (Furse et al., 2019; Rahman et al., 2021), and a study by Lai et al. found decreased SM levels in women with GDM to be associated with increased risk of developing type 2 diabetes (Lai et al., 2020). Sphingomyelins may play an indirect role in impaired insulin signaling, as increased levels of ceramides, a sphingolipid that can be derived from sphingomyelins, have been reported to inhibit the insulin signaling pathway, while also disturbing mitochondrial respiration (Roszczyc-Owsiejczuk & Zabielski, 2021). Inhibition of sphingomyelin synthase, the enzyme that converts ceramides to sphingomyelin, has been found to trigger ceramide buildup, which then inhibits insulin signaling. However, the connection between sphingomyelins and insulin resistance is not well understood and is controversial due to some conflicting results, so further research is needed (Roszczyc-Owsiejczuk & Zabielski, 2021).

This study highlights metabolites associated early detection of GDM, including hypoxanthine, alpha-hydroxybutyrate, xanthine. All these metabolites have biological plausibility to connect with GDM and support previous metabolic evidence showing that these metabolites are associated with pre-diabetic conditions, insulin resistance, and type 1 and type 2 diabetes mellitus (Cobb et al., 2013; Gall et al., 2010; Ishikawa et al., 2013; Sheikh-Ali et al., 2008; Yamamoto et al., 1997). However, it is important to acknowledge several limitations that impact our ability to confirm these metabolites as markers of GDM. First, it is commonly accepted that a biomarker for clinical use needs sensitivities and specificities ≥0.9. However, AUCs in the category of “excellent” diagnostic accuracy (AUC >0.9) are ideally needed to get to these high values. In our case our best diagnostic accuracy was found for a four-metabolite panel at the level of “very good” diagnostic accuracy (AUC between 0.8 and 0.9). Due to limitations of the use of individual metabolites as biomarkers because many have associations with a variety of disease conditions, we have focused primarily on our multimarker panel that was analyzed. The lack of capacity to accurately propose cut points of abnormality because our metabolomics analyses were not based on the absolute quantification of metabolite concentrations was another limitation. The study is limited to the specific targeted metabolites of the metabolomics assays utilized. An untargeted metabolomics approach may have yielded more metabolites that were different between groups. Lastly, the ASA-24 is subject to limitations, as it relied on self-administration and therefore may not accurately reflect participants’ dietary intake.

This project is novel due to its comprehensive metabolomics analysis of first trimester metabolites that spans from polar metabolites to complex lipids collected from a RCT. Most studies have addressed later trimesters after GDM has been diagnosed or use one metabolomics assay that detects either polar or non-polar compounds (Q. Chen et al., 2018). Other notable strengths of the study include the use of samples from an RCT, reducing bias by utilization of a double-blind method. Based on dietary intake analyses, it appears that the metabolomic differences between groups were unlikely to be diet-related. However, the dietary data in combination with using participants paired by age, BMI, ethnicity, and treatment group, strengthens the ability to detect differences due to GDM. Samples were attained from two sites in opposite areas of the United States, allowing the results to be a more accurate representation of a larger population.

## Conclusion

The metabolomics profiling conducted in this study shows that plasma metabolites involved in purine degradation, insulin resistance, and fatty acid oxidation were the most altered in early pregnancy in connection with subsequent diagnosis of GDM. The top three single potential metabolites associated with prediction of GDM were xanthine, hypoxanthine and alpha-hydroxybutyrate. A panel composed of four metabolites is proposed to potentially predict GDM from first trimester blood collection. Future studies are needed to validate these compounds as potential biomarkers of GDM and to propose cut points of abnormality.

## Supplementary Material

1760541_OR

1760541_OR_Table 1

1760541_Sup_file-2

1760541_Sup_file-3

## Figures and Tables

**Table 1.** Subject characteristics and baseline cardiometabolic risk factors for non-GDM and GDM groups*

Subject characteristics and biochemical tests	Non-GDM (n= 34)		GDM (n= 34)*		

	Mean	SD	Mean	SD	p-value
***Age (y)***	31.5	5.7	31.5	± 4.9	0.98
***Body mass index (kg/m^2^)***	33.6	5.2	34.8	± 5.2	0.34
***Hispanic, %***	44.1%		38.2%		0.56

***Glucose (mg/dL)***	88.7	8.1	91.1	10.2	0.44
***Insulin (μU/mL)***	20.1	13.3	26.0	10.0	0.05
***HOMA-IR***	4.5	3.3	5.5	2.7	0.06
***Total Cholesterol (mg/dL)***	193.6	40.8	203.6	48.7	0.46
***LDL Cholesterol (mg/dL)*** ¥	95	27.1	104.0	42.0	0.49
***HDL Cholesterol (mg/dL)*** †	66.2	15.6	59.9	15.1	0.04
***Triglycerides (mg/dL)***	162.7	81.7	204	89.8	0.03
***C-Peptide (ng/mL)***	3.0	1.4	3.5	1.5	0.06
***Leptin (μg/L)***	58.8	16.6	67.4	25.7	0.30

Data presented as mean ± SD unless otherwise stated.

* One participant for the GDM group did not have cardiometabolic risk factor data

¥ Friedewald LDL Cholesterol

† HDL Chol, Direct

*Abbreviation:* Non-GDM, non-gestational diabetes mellitus; GDM, gestational diabetes mellitus; SD, standard deviation.

**Table 2:** Significantly different metabolites between GDM cases and healthy controls at first trimester.

Metabolite	Metabolite Class	Non-GDM		GDM		p-value^¥^	AUC^†^	Diagnostic accuracy

		Mean	SD	Mean	SD			

***Hypoxanthine*** *	Purines, pyrimidines, nucleotides, nucleosides	173211.7	91556.5	243337.003	123891.0	0.00008	0.73	Good
***alpha-hydroxybutyrate***	Carboxylic acids	1573737.3	559803.0	2017387.7	834561.3	0.0008	0.72	Good
***xanthine***	Purines, pyrimidines, nucleotides, nucleosides	64433.9	25364.3	84059.8	31434.3	0.001	0.70	Good
***SM C14:0***	Sphingolipids	232746436	23385990.4	211523493	26747823.8	0.005	0.78	Good
***SM C15:0***	Sphingolipids	61120821.5	12691406.7	52816697.3	14303954.9	0.011	0.70	Good
***uridine***	Purines, pyrimidines, nucleotides, nucleosides	519149.5	172358.8	610840.5	211832.8	0.021	0.65	Sufficient
***beta-hydroxybutyrate***	Ketone bodies	487780.8	359071.6	749600.8	726367.25	0.024	0.66	Sufficient
***SM C16:1***	Sphingolipids	273094637	30963123.3	252692544	40031560.5	0.032	0.68	Sufficient
***kynurenine***	Tryptophan metabolism	77915.8	104996.0	110934.4	117921.7	0.032	0.59	Not-sufficient
***nicotinamide***	Tryptophan metabolism	38018.5	15224.6	81531.0	225036.6	0.043	0.61	Sufficient
***SM C22:0***	Sphingolipids	1285905343	117868787.9	1187471739	185105947.7	0.043	0.69	Sufficient
***Palmitoylcarnitine***	Acylcarnitines	424578.3	169459.8	513203.1	194186.1	0.046	0.63	Sufficient
***Malonylcarnitine***	Acylcarnitines	50728.8	46707.6	68094.0	52181.2	0.048	0.61	Sufficient

* Values are in peak area

¥ Metabolites ranked by raw p-values.

† AUCs were calculated from ROC curve analysis and were classified as “not useful”, “not sufficient”, “sufficient”, “good”, “very good” or “excellent”, respectively, if were < 0.5, between 0.5-0.6, 0.6–0.7, 0.7–0.8, 0.8–0.9 or 0.9-1.0, respectively.

Note: Hypoxanthine detected with the primary metabolomics assay is shown. Hypoxanthine was also detected with the Aminomics assay and had a similar result (p<0.001)

Abbreviations: Non-GDM, non-gestational diabetes mellitus; GDM, gestational diabetes mellitus; SD, standard deviation, AUC, area under the curve;
