# Supplementary material for: Metabolites Involved in Purine Degradation, Insulin Resistance, and Fatty Acid Oxidation are Associated with Prediction of Gestational Diabetes in Plasma
Source: Metabolomics. Author manuscript; Available in PMC 2022 Nov 27. (PMC8741304; doi:10.1007/s11306-021-01857-5)
Supplement: 1760541_OR [file NIHMS1760541-supplement-1760541_OR.docx]

**Online Resource**

**Metabolites Involved in Purine Degradation, Insulin Resistance, and Fatty Acid Oxidation are Associated with Prediction of Gestational Diabetes in Plasma**

Lauren E. McMichael^a^, Hannah Heath^a^, Catherine M. Johnson^a^, Rob Fanter^b,c^, Noemi Alarcon^d,e^, Adilene Quintana-Diaz^d,e^, Kari Pilolla^a,e^, Andrew Schaffner^e,f^, Elissa Jelalian^g^, Rena R. Wing^g^, Alex Brito^h,i^, Suzanne Phelan S^d,e^, Michael R. La Frano^a,c,e,#^

^a^Department of Food Science and Nutrition, California Polytechnic State University, San Luis Obispo, CA

^b^College of Agriculture, Food and Environmental Sciences, California Polytechnic State University, San Luis Obispo, CA

^c^Cal Poly Metabolomics Service Center, California Polytechnic State University, San Luis Obispo, CA

^d^Department of Kinesiology and Public Health, California Polytechnic State University, San Luis Obispo, CA

^e^Center for Health Research, California Polytechnic State University, San Luis Obispo, CA

^f^Department of Statistics, California Polytechnic State University, San Luis Obispo, CA

^g^Warren Alpert Medical School at Brown University Department of Psychiatry and Human Behavior, Providence, RI

^h^Laboratory of Pharmacokinetics and Metabolomic Analysis. Institute of Translational Medicine and Biotechnology. I.M. Sechenov First Moscow Medical University, Moscow, Russia

^i^World-Class Research Center "Digital Biodesign and Personalized Healthcare", I.M. Sechenov First Moscow State Medical University, Moscow, Russia.

Lauren E. McMichael and Hannah Heath contributed equally to warrant co-first authorship.

# Corresponding Author: Michael R. La Frano, PhD, RD, Department of Food Science and Nutrition, Center for Health Research, Cal Poly Metabolomics Service Center, California Polytechnic State University, San Luis Obispo, 1 Grand Ave, San Luis Obispo, CA 93407. Email: [mlafrano@calpoly.edu](mailto:mlafrano@calpoly.edu)

**
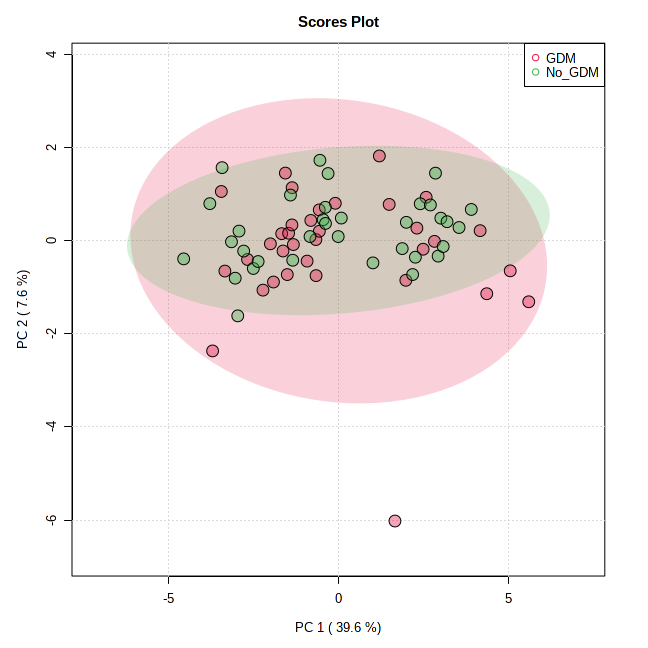
**

**Online Resource Figure 1.** Scores for a two-component principal component analysis for assessment

of GDM versus non-GDM group discrimination.

**
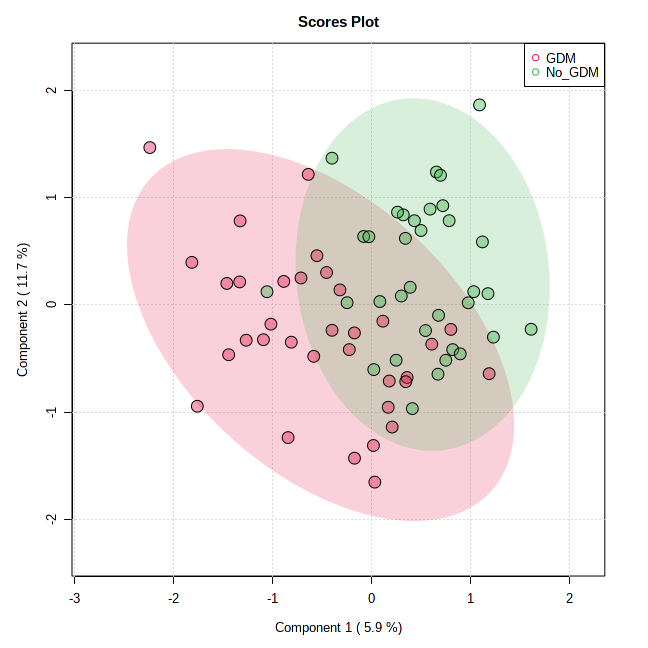
**

**Online Resource Figure 2.** Scores for a two-component partial least squares-discriminant analysis

model to visualize GDM versus non-GDM group discrimination.

**A**


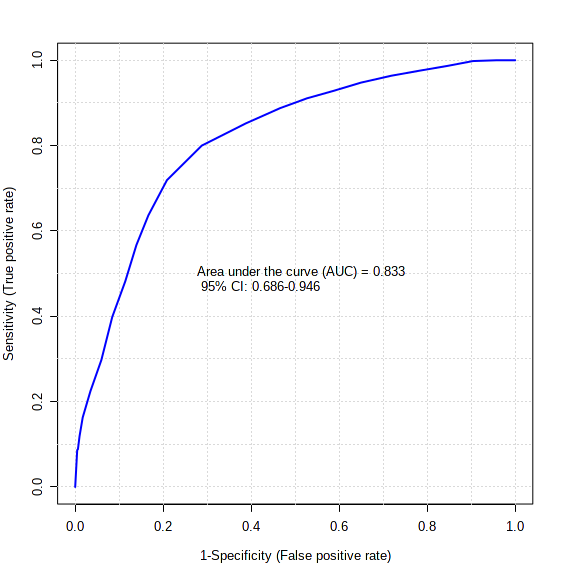


**B**


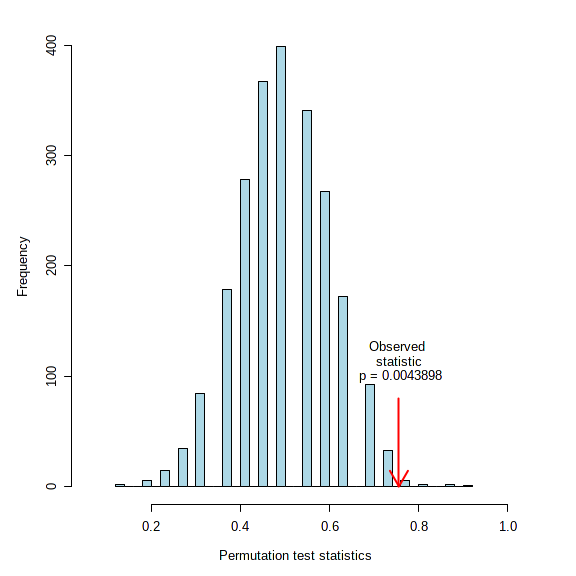


**Online Resource Figure 3.** (A) Receiver Operating Characteristic (ROC) curve analysis for a panel of four metabolites: Sphingomyelin 14:0, hypoxanthine, alpha-hydroxybutyrate, and xanthine; (B) Predictive accuracy using 100 permutations was used to measure performance.

**Online Resource Table 1.** Data summary for all detected metabolites available in separate Excel file

**Online Resource Table 2.** Dietary intake data.

| **Dietary Item** | **Non-GDM** | | **GDM** | |  |
| --- | --- | --- | --- | --- | --- |
|  | ***Mean*** | ***SD*** | ***Mean*** | ***SD*** | ***p-value*** |
| **Carbohydrates. %Kcal** | 50.3 | 9.5 | 49.2 | 6.6 | 0.70 |
| **Fat, %Kcal** | 34.1 | 8.2 | 34.2 | 4.9 | 0.90 |
| **Protein, %Kcal** | 17.6 | 4.2 | 17.6 | 3.3 | 0.86 |
| **Calories, Kcal** | 1705.6 | 528.3 | 1842.1 | 493.4 | 0.24 |
| **Total fat, g** | 65.3 | 30.3 | 69.9 | 22.2 | 0.25 |
| **Saturated fat, g** | 21.8 | 10.3 | 23.4 | 8.1 | 0.24 |
| **Monounsaturated fat, g** | 23.4 | 10.9 | 25.0 | 8.8 | 0.31 |
| **Cholesterol, mg** | 246.8 | 142.0 | 300.0 | 178.3 | 0.21 |
| **Sodium, g** | 2956.1 | 953.0 | 3368.7 | 866.7 | 0.045 |
| **Carbohydrates, g** | 214.9 | 81.6 | 227.4 | 68.8 | 0.39 |
| **Sugar, g** | 101.2 | 59.7 | 98.5 | 41.2 | 0.93 |
| **Fiber, g** | 16.6 | 7.8 | 14.7 | 5.5 | 0.25 |
| **Protein, g** | 72.8 | 21.1 | 80.0 | 25.5 | 0.24 |
| **Potassium, mg** | 2350.9 | 704.7 | 2273.8 | 824.8 | 0.50 |
| **Iron, mg** | 59.1 | 57.3 | 49.7 | 40.3 | 0.48 |
| **Vitamin D, µg** | 16.4 | 5.3 | 15.8 | 7.6 | 0.78 |
| **Vitamin A, µg RAE** | 1927.8 | 599.6 | 1991.1 | 760.5 | 0.36 |
| **Folate, µg** | 1370.8 | 455.5 | 1293.2 | 594.0 | 0.80 |
| **Vitamin B12, µg** | 15.6 | 7.1 | 29.5 | 91.4 | 0.24 |

**Data presented represents Daily Total Nutrients from Foods and Supplements (TNS)*

*Abbreviation:* Non-GDM, non-gestational diabetes mellitus; GDM, gestational diabetes mellitus; SD, standard deviation; RAE, retinol activity equivalents
